# Novel Determination of Functional Groups in Partially Acrylated Epoxidized Soybean Oil

**DOI:** 10.3390/molecules29194582

**Published:** 2024-09-26

**Authors:** Olga Gómez-de-Miranda-Jiménez-de-Aberasturi, Javier Calvo, Ingemar Svensson, Noelia Blanco, Leire Lorenzo, Raquel Rodriguez

**Affiliations:** 1 Tecnalia R&I, Basque Research and Technology Alliance (BRTA), Parque Tecnológico de Alava 01510, Leonardo da Vinci 11, 01510 Vitoria-Gasteiz, Spain; ingemar.svensson@tecnalia.com (I.S.); noelia.blanco@tecnalia.com (N.B.); leire.lorenzo@tecnalia.com (L.L.); raquel.rodriguez@tecnalia.com (R.R.); 2 Center for Cooperative Research in Biomaterials (CIC BiomaGUNE), Basque Research and Technology Alliance (BRTA), Paseo de Miramon 194, 20014 Donostia-San Sebastián, Spain; jcalvo@cicbiomagune.es

**Keywords:** soybean oil, epoxidized intermediate, acrylated monomer, mass spectrometry, electrospray ionization, ultra-high-performance liquid chromatography

## Abstract

The acrylation degree of vegetable oils plays a relevant role in determining the mechanical properties of the resulting polymers. Both epoxide and acrylate functionalities participate in polymerization reactions, producing various types of chemical bonds in the polymer network, which contribute to specific properties such as molecular size distribution, crosslinking degree, and glass transition temperature (Tg). The accurate identification of epoxide and acrylated groups in triglyceride molecules helps to predict their behavior during the polymerization process. A methodology based on analytical spectrometric techniques, such as direct infusion, mass spectrometry with electrospray ionization, and ultra-high-performance liquid chromatography, is used in combination with FTIR and ^1^H NMR to characterize the epoxy and acrylic functionalities in the fatty chains with different numbers of carbon atoms of partially acrylated triglycerides obtained by a non-catalytic reaction.

## 1. Introduction

There is a global shift from using petroleum-based products to bio-based ones that are obtained from renewable resources like biomass. This is composed from organic matter such as vegetable or animal oils, garbage, waste, lignin, and cellulose [1]. Utilizing bio-based products from biomass, such as bio-epoxides from seed oils, bio-polyols, and bio-based acrylates or polyesters, is crucial for introducing biogenic carbon into the product life cycle, ultimately leading to a reduction in the carbon footprint by sequestering atmospheric carbon dioxide through photosynthesis [2]. Using biomass-derived raw materials for various polymer applications, such as coatings, adhesives, elastomers, and composites, is the most direct and effective approach. Examples include components made from seed oils or vegetable oils, bio-based polymers, UV-cured systems, polymers from recycled residues, and byproducts from secondary bio-based streams. The commitment to developing more sustainable products and processes constitutes a necessity for continuous innovation for the future industrial manufacturers [3]. In this context, soybean oil (SO) is a great renewable resource that is abundant, versatile, and low cost. It was employed to obtain acrylated monomers that have the necessary reactivity to synthesize new bio-based polymers as a sustainable alternative to traditional petroleum-based platform molecules.

Acrylated oils constitute a very promising alternative to be employed in diverse coating applications. The lower viscosity of these materials compared to conventional epoxy acrylates eliminates the need for their reduction with a reactive diluent [4]. They are primarily flexible and possess an aliphatic acrylic backbone, which can significantly improve the UV coating flexibility and adhesion of the polymer [4,5]. Special interest has been paid to UV-curable coatings, where acrylated oils react by means of UV-initiated free radical polymerizations in the presence of specific photoinitiators. As an example, new photocurable biocompatible liquid resins have been developed for 3D stereolithography-based bioprinting [6,7]. Acrylated coatings have also been developed to reduce the moisture sensitivity and permeability of bio-based films [8].

The acrylation degree of the oil is an important factor that is very relevant for the mechanical properties of the polymer. In this way, vegetable oils are not always fully acrylated to obtain monomers with specific characteristics. Boucher et al. [9] epoxidized and partially acrylated linseed oil and copolymerized it with (3,4-dihydroxyphenetyl)-acrylamide and N,N-dimethyl acrylamide to develop a coating for corrosion protection. A series of partially acrylated vegetable oils with different functional groups was synthesized and further polymerized with styrene [10]. Several methyl esters of C16 to C24 derived from camelina oil and linseed oil were epoxidized, fully or partially acrylated, and then polymerized in emulsion with various amounts of bio-based derivatives (5–30 wt.% in monomer mixture) to obtain polymeric latexes for coating formulations [11].

Typically, acrylated epoxidized oils are obtained by adding acrylic acid (AA) to epoxidized vegetable oils (ESO if they are obtained from SO), which are more reactive than the original raw materials. The commercial production of epoxidized oils is carried out with homogeneous acidic catalysts, carboxylic acids (principally acetic or formic acids), and an aqueous hydrogen peroxide solution, usually in concentrations between 30 and 50% *w*/*w* [12].

The acrylation process is usually carried out with an excess of AA, hydroquinone as a free radical inhibitor, and different catalysts. 1,4-diazobicyclooctane with temperatures up to 95 °C and times up to 11 h has been used [13]. Acrylated epoxidized soybean oil (AESO) was also synthesized using triphenylphosphine oxide as the catalyst and a temperature of 120 °C for 6 h, achieving a conversion of 95% [5]. Triethylamine was also considered as an efficient catalyst for the acrylation of epoxidized oils. In this case, a temperature of 80 °C and 8 h were employed to achieve an average conversion of the epoxidized groups of 70% [14]. Although they are common catalysts, they need to be handled with extra precautions due to their high oral and inhalation toxicity [15]. A solution of 40–60% chromium (III) 2-ethylhexanoate in a mixture of di (heptyl, nonyl, undecyl) phthalates) was demonstrated to be one of the most effective catalysts in the acrylation of vegetable oils. Complete acrylation of camelina oil with AA was obtained at 80 °C with 2% of this catalyst in 12 h [16].

Catalytic acrylation reactions offer several advantages, such as higher reaction rates at lower temperatures, but they also show some limitations. Once the acrylation process is finished, it is essential to purify the product to remove any traces of remaining AA and catalyst, which may negatively affect the product’s further applications. Spent catalyst residues must be properly handled.

Meléndez et al. [17] studied the synthesis of an oligo-acrylated product from soya oil and acrylic acid without using any catalyst, which was obtained in 86% yield after 12 h at 120 °C. The acrylated monomers studied in this paper were obtained without the use of any catalyst to avoid the generation of any residual stream. The reactant ratio and operational conditions were selected to obtain a product with a high acrylation degree with lower reaction times to avoid the formation of undesired acrylic oil auto-polymerization.

On the other hand, the application of different analytical technologies is crucial for a deep analysis of the molecular structure of acrylated molecules. The most commonly used methods to characterize acrylated monomers include FTIR spectroscopy, which qualitatively describes the different functional groups present in triglyceride molecules, and ^1^HNMR to calculate the average inclusion degree of AA in the epoxidized fatty chains. Chemical titrations such as the iodine value (IV) and the oxirane content (COOe) are used to determine the double bond conversion in the epoxidation reaction and the remaining epoxide presence after the acrylation reaction, respectively [18]. A better understanding of partially acrylated structures using mass spectrophotometric techniques would permit a visual identification of the different functionalities present in fatty chains of various lengths and original unsaturation degree. Triglycerides with no functionalities, those with a single functionality, and molecules with more than one acrylate group could be distinguished, which is interesting for controlling polymerization reactions.

Kuki et al. [19] employed matrix-assisted laser desorption ionization and electrospray ionization mass spectrometry (MALDI-MS and ESI-MS) for the characterization of epoxidized soybean and linseed oils. These techniques allowed for the identification of different epoxidized triglyceride (TG) mass spectral peak series and the number of epoxide groups in the products without any complicated and time-consuming sample preparation.

In this study, different spectroscopy techniques, such as direct infusion, mass spectrometry with electrospray ionization, and ultra-high-performance liquid chromatography were combined with FTIR and ^1^HNMR to define a methodology for the characterization of the epoxy and acrylic functionalities in fatty chains with different numbers of carbon atoms.

## 2. Results and Discussion

First, SO was epoxidized with acetic acid and hydrogen peroxide (50% *w*/*w*) using H_2_SO_4_ (96%) as the acid catalyst. The purification process of the intermediates did not involve the use of any hazardous organic solvents, since impurities, unreacted materials, and catalyst residues were removed from the epoxidized intermediate through 4 washes with a warm brine solution (60 °C).

The epoxidized samples presented a COOe of 6.6 g O/100 g, and unsaturation degree of 8 g I_2_/100 g. These values were determined by measuring the oxirane content and the IV of ESO. According to this, the oxirane yield calculated for the ESO intermediate was 88%.

The FTIR spectra of the samples demonstrated the development of distinctive peaks for the epoxidized products. A peak at 830 cm^−1^, characteristic of the epoxide group, was observed, which evidenced that the reaction took place. Other representative peaks related to the fatty unsaturations =C–H at 3000 cm^−1^ and C=C at 1651 cm^−1^ appeared in the spectrum of SO but disappeared in the ESO spectrum, indicative that the internal double bonds of SO reacted with the peracid.

**Figure 1:**
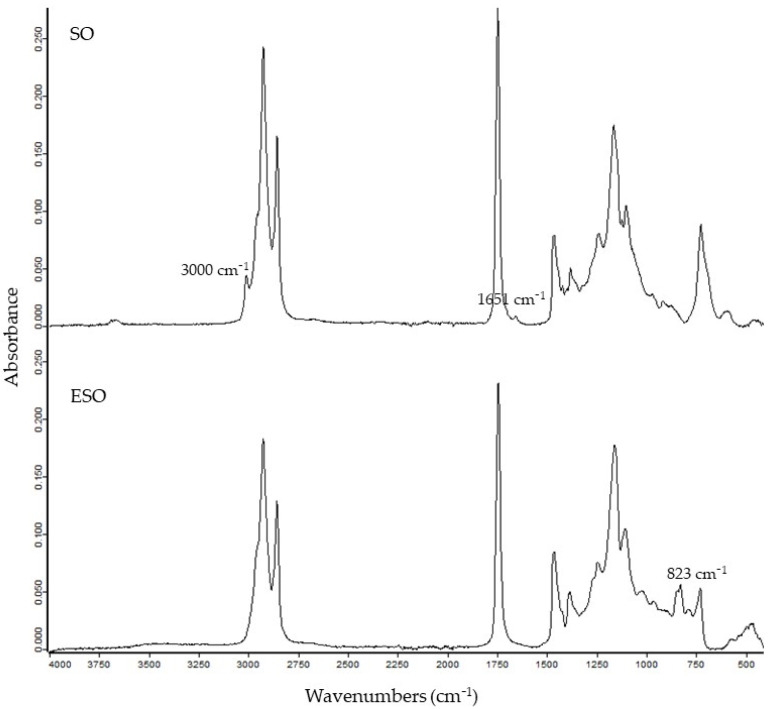
FTIR spectra of SO and ESO.

Table 1 displays the most relevant peaks found in the spectra of SO and ESO. It is noted that no peaks were present at wavelengths between 3300 and 3500 cm^−1^, which are typical of -OH groups. This confirmed that no undesired reactions for producing glycols occurred. (Figure 1). 

**Scheme 1:**
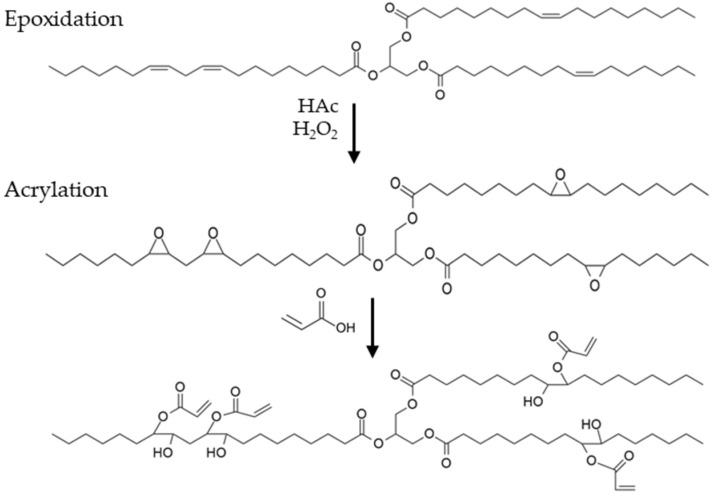
Acrylation of SO in two steps through ESO intermediates.

It is well known that the oxirane rings of ESO consist of tensioned structures that easily react with nucleophiles such as alcohols, amines, or protic acids. These molecules interact with the electrophilic carbons of the epoxide rings, polarizing them, breaking the initial bonds, and creating new ones as ester and hydroxyl functionalities (Scheme 1).

In the second step, the epoxidized intermediates were reacted with AA to produce acrylated monomers. A slight excess of acid was used (RM AA/epoxide groups 1.1:1) along with hydroquinone (0.2% *w*/*w*) to prevent the auto-polymerization of AA.

Additionally, the acrylation process was carried out without a catalyst to avoid the generation of effluents containing spent catalyst, which are difficult to recover. AESO was obtained by controlling both the reagent ratio and temperature. Previous references described that the synthesis of an acrylated oligomer could be performed at 120 °C without generating toxic effluents containing spent catalyst [17]. Here, the formation of AESO was maintained for 4 h and monitored using FTIR spectroscopy to track the evolution of the different molecules that were being synthesized. The remaining epoxide groups were determined by the oxirane content measurement, which gave a value of 0.81 g O/100 g.

**Figure 2:**
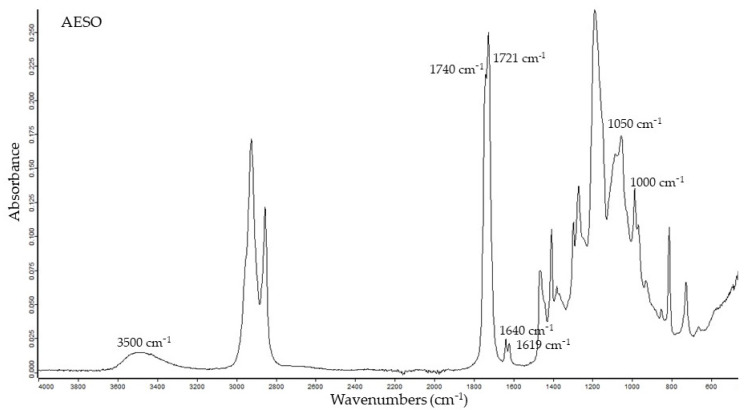
FTIR spectra of thermally obtained AESO without catalyst.

It was observed that longer reaction times led to undesired auto-polymerization of AA and/or AESO molecules, evidenced by a decrease in the acrylic peak length (Figure 2). These could limit the overall product yield despite full conversion. When the oxirane ring is opened with AA, it produces an AESO molecule with a secondary hydroxyl group. However, this secondary hydroxyl group can further esterify with another acrylic acid and then produce water. Alternatively, water can react with other epoxide rings, yielding diols as a byproduct [17].

The disappearance of peaks at 823–830 cm^−1^ corresponding to epoxy groups indicated high reaction conversion. The remaining oxirane functionalities in the product could not be observed in the spectrum with enough precision. Additionally, new peaks attributed to acrylate groups (CH2=CH–COO–) appeared at 1619 cm^−1^. The signals at 3400–3500 cm^−1^ corresponding to the formation of hydroxyl groups confirmed the opening of oxirane rings to form acrylate groups and alcohols. Other notable peaks included the C=O stretching vibration of acrylate ester groups at 1721 cm^−1^ close to the triglyceride ester peak at 1740 cm^−1^. Other relevant peaks were C–O stretching at 1000 cm^−1^, =C–H bending (twisting) at 1050 cm^−1,^ and the trans and cis C=C stretching vibration at 1640 cm^−1^, all of which demonstrated the attachment of acrylate groups to fatty acid chains. The principal FTIR signals are listed in Table 2.

**Figure 3:**
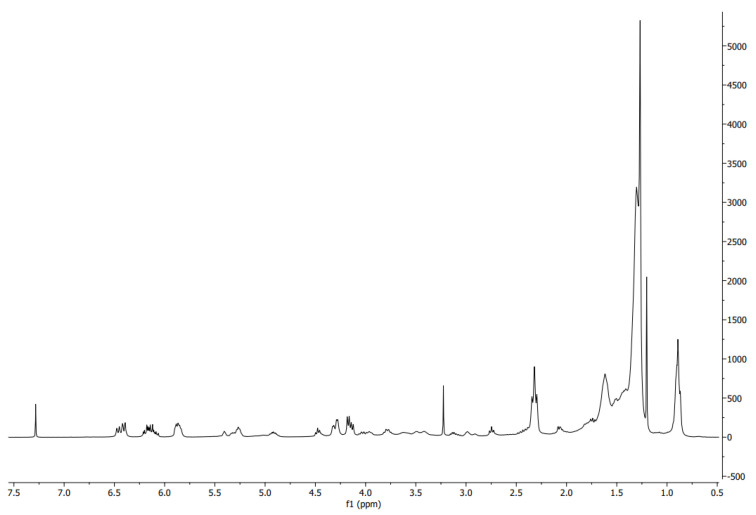
^1^H NMR spectra of the thermally synthesized AESO.

Figure 3 shows the ^1^H NMR spectrum of thermically acrylated epoxidized soybean oil. The two sets of peaks from 4.0 to 4.4 ppm are produced by the four methylene hydrogen atoms attached to the glycerol center. The peak at 2.3 ppm is produced by the six methylene hydrogen atoms alpha to the carbonyl groups. The peak areas of the four methylene hydrogens in glycerol were used as the internal standard to determine the number of acrylates per triacylglycerol by comparing with the peak areas from the three acrylate protons (5.7–6.6 ppm). Considering an equivalent methodology to the ones described by Zhang et al. [20] and Su et al. [21], the acrylated molecule/TG ratio was determined to give a value of 2.1 using the following formula:

(1) $$n=\frac{\mathrm{Area}\;\left(\mathrm{vinyl}\;\mathrm{protons}:5.7\text{–}6.6\;\mathrm{ppm}\right)}{\mathrm{Area}\;\left(\mathrm{glycerol}\;\mathrm{methylens}:4.4\text{–}4.4\;\mathrm{ppm}\right)}\;\cdot \;\frac{4}{3}$$

The authors defined an initial number of double bonds per SO molecule as 4.08. Therefore, the conversion of double bonds to acrylates was calculated to be 51%.

The signals of the protons [–CHOCH–] corresponding to the remaining epoxide groups in the acrylate monomers at 2.9 ppm could not be distinguished with high precision. Additional techniques were necessary for a more detailed analysis of the different functionalities present in the product.

A specific methodology based on electrospray ionization techniques was developed to clearly distinguish AESO molecules with similar masses but different geometries, which can cause different steric effects and reactivity and affect in a different way further polymerization processes. These technologies are favored over others due to their gentle ionization process, ensuring intact ionization and analysis of samples. They are most suitable for measuring lipid substances and can efficiently identify changes in their different structures, such as unsaturated, epoxidized, or acrylated functionalities. Additionally, ultra-high-performance liquid chromatography and mass spectrometry detection are used before ionization to improve detection. These steps ensure that the ionization of the main substances does not interfere with the ionization and detection of less abundant ones, also known as ion suppression.

Three different types of samples were analyzed by mass spectrometry techniques: (1) unreacted SO; (2) ESO intermediate, and (3) AESO sample obtained by a non-catalytic reaction. Two different approaches were carried out for the characterization of the functional groups present in the molecules.

- Method 1: Direct sample infusion and mass spectrometry detection with an electrospray source and high-resolution time-of-flight analyzer (DI-ESI-TOF-MS).
- Method 2: Ultra-high-performance liquid chromatography and mass spectrometry detection with an electrospray source and high-resolution time-of-flight analyzer (UHPLC/ESI-TOF-MS).

**Figure 4:**
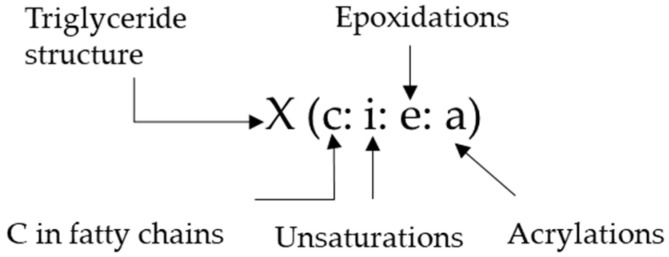
Nomenclature used in mass spectrometry analysis.

To make the identification of unsaturations, epoxidations, and acrylated groups easier, the diverse TG structures were named as shown in Figure 4.

**Figure 5:**
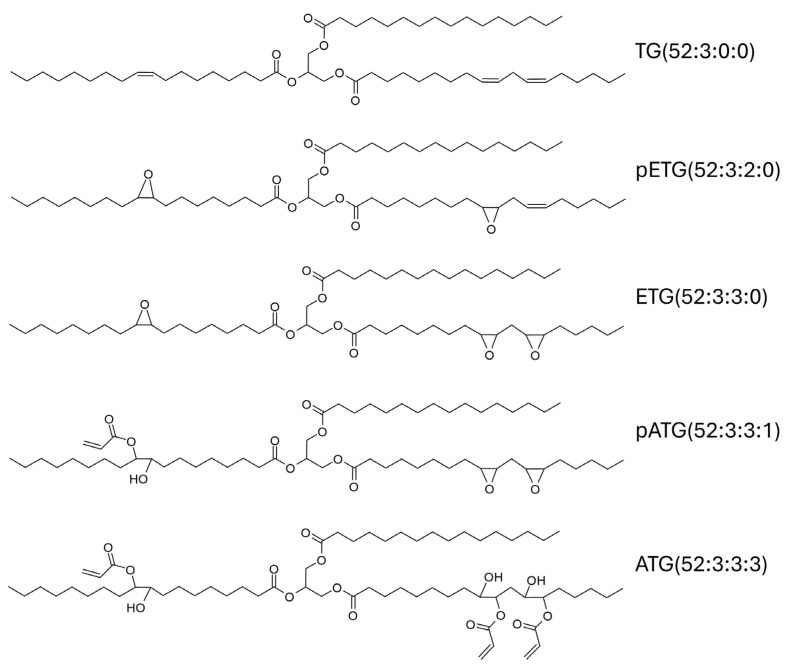
Hypothetical structures of a partially acrylated TG of 52C with 3 original unsaturations and with different degree of epoxidation and acrylation.

The possibilities of the TG distribution analyzed are very broad. Molecules of different fatty chain lengths, unreacted TGs (TGs), fully epoxidized TGs (ETG), partially epoxidized TGs (pETG), completely acrylated TGs (ATG), and partially epoxidized and acrylated TGs (pATG) could be detected. In Figure 5, an example of the nomenclature used for the determination of sample molecules is shown.

**Figure 6:**
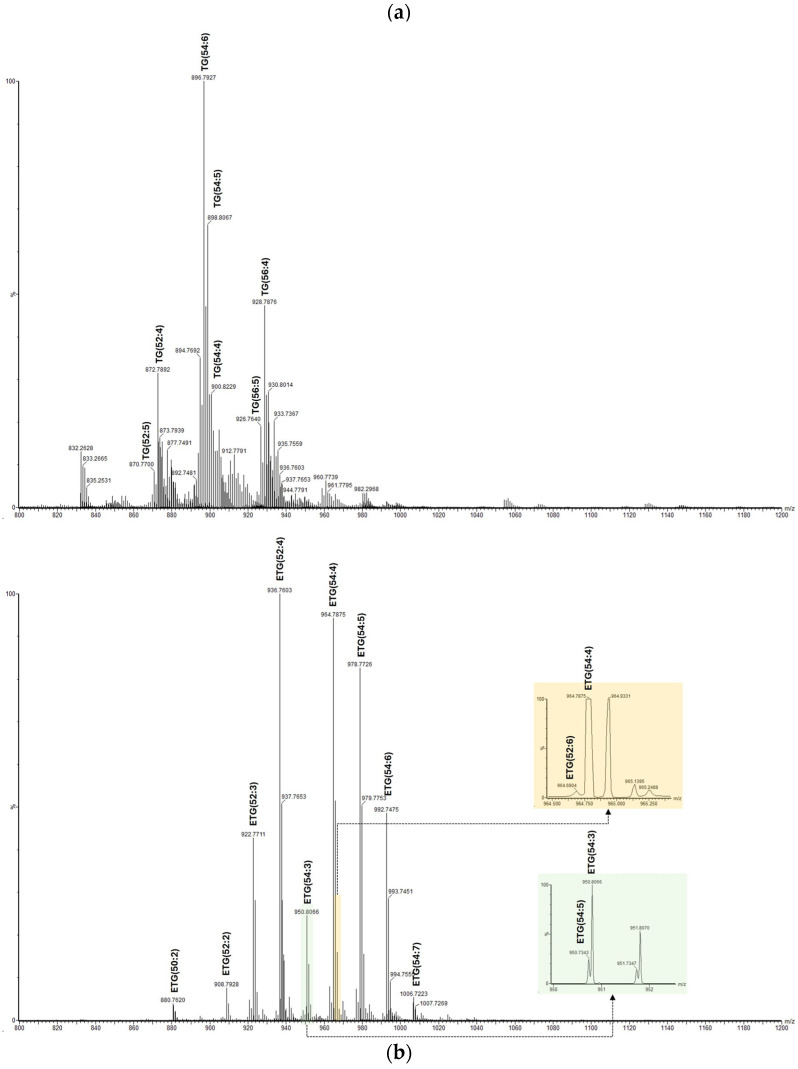
DI-ESI-TOF-MS spectra of SO (**a**) and ESO samples (**b**).

It was evidenced that by using DI-ESI-TOF-MS (method 1), no epoxidation or acrylation functionalities appeared in the SO sample (Figure 6a) due to the absence of signals corresponding to these structures in comparison to the corresponding commercial extracts. Different fully epoxidized molecules (even for the less abundant TGs) were found in the ESO intermediates thanks to the high resolution of the technique (Figure 6b).

**Figure 7:**
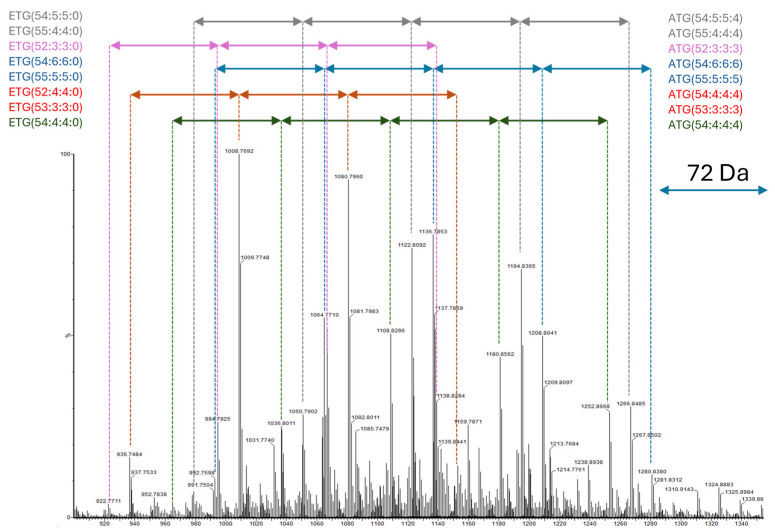
DI-ESI-TOF-MS spectra of thermally synthesized AESO. Identification of different acrylation series: ETG; pETG; ATG.

In the AESO sample synthesized without using any catalyst, epoxidized and acrylated functionalities were found in TGs with different chain lengths. This revealed the uncomplete conversion of the reaction (Figure 7). Both functionalities were more clearly observed with this analytical technique than with others.

Taking into consideration a mass difference Δ*m*/*z* = 72 between the epoxy and the acrylate groups, a series of TGs with several acrylation degrees was identified considering the mass differences between the epoxidized/acrylated groups.

Analyzing more in detail the series (54:4), in which the assignment was unambiguous, it was possible to calculate the epoxidation/acrylation ratio, assuming that all triglycerides, having very similar structures, ionize in a similar way in the mass spectrometer.

If m is defined as the total number of functionalized triglyceride unsaturations, either as epoxy or as acrylate, the total concentration of this group is given by the following formula:

(2) $$C_{\mathrm{func}\;\left(m,n\right)}=C_{\mathrm{total}\;\left(m,n\right)}\;\cdot \;m$$

Similarly, if n is defined as the number of acrylate groups in a sample, the concentration of this group can be calculated as follows:

(3) $$C_{\mathrm{acrylated}\;\left(m,n\right)}=C_{\mathrm{total}\;\left(m,n\right)}\;\cdot \;n$$

And the concentration of the epoxy group in the sample is defined as follows:

(4) $$C_{\mathrm{epoxi}\;\left(m,n\right)}=C_{\mathrm{total}\;\left(m,n\right)}\;\cdot \;\left(m-n\right)$$

Thus, the acrylation percentage %AC of a specific triglyceride defined by m functionalized unsaturations and n acrylate ones is determined by the following equation:

(5) $$\%\mathrm{Ac}=100\times \frac{\sum_{n=0\;}^{m}n\cdot C_{\mathrm{total}\;\left(m,n\right)}\;}{\sum_{n=0\;}^{m}m\cdot C_{\mathrm{total}\;\left(m,n\right)}\;}$$

On the other hand, if we define ionizability obtained by mass spectrometry, i, as the ratio between the intensity (Int) obtained from a signal and its relationship to the concentration of the analyte, assuming that the ionizability of fully epoxidized, partially acrylated, or fully acrylated triglycerides is similar and proportional, the acrylation percentage can be defined as follows:

(6) $$\%\mathrm{Ac}=100\times \frac{\sum_{n=0}^{m}n\cdot {\mathrm{Int}}_{\left(m,n\right)}\;}{m\cdot \;\sum_{n=0\;}^{m}{\mathrm{Int}}_{\left(m,n\right)}\;}$$

**Figure 8:**
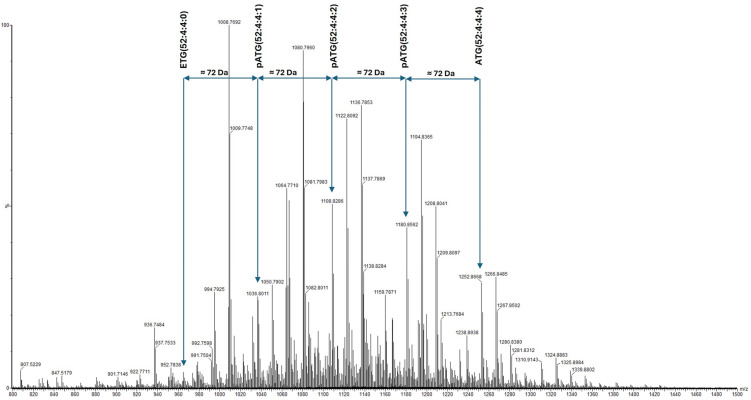
DI-ESI-TOF-MS spectra of AESO synthesized without catalyst—series assignation ETG(54:4:4:0); ATG(54:4:4:4). Total and relative intensities.

It is worth mentioning that making an unequivocal assignment between different lipids is quite difficult since the increase in mass due to one more carbon in a fatty acid and the presence of an epoxy group offer very similar signals (*m*/*z* 13.98, 14.02). However, it is possible to estimate the percentage of ionization based on the assigned signals corresponding to unique structures and assuming that the ionizability of these structures is similar concerning the number of epoxy/acrylate groups. Using the intensities for the different components Int (m,n) obtained in the ETG(54:4:4:0); ATG(54:4:4:4), in relation to the acrylation number (n) and the number of total epoxides groups (m) (Figure 8 and Table 3), it could be confidently approximated that the percentages of acrylation with respect to the epoxidized groups were around 61.1% (ec 7) and 53.7% of the initial double bonds of SO (taking in consideration that the oxirane yield calculated for the ESO intermediate was 88%).

The results obtained fit with the corresponding ones obtained by ^1^H NMR, taking into consideration that the oxirane yield calculated for the ESO intermediate was 88%.

**Figure 9:**
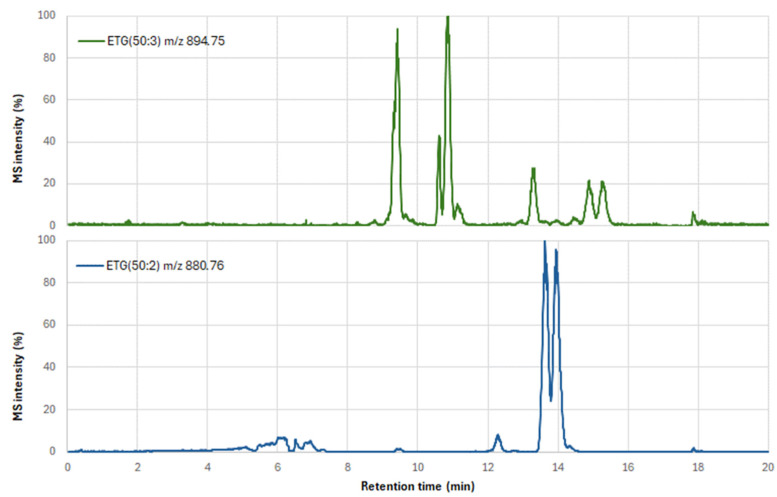
Extracted-ion chromatograms (XIC) at *m*/*z* 880.76 and 894.75 corresponding to the structures ETG(50:2) and ETG(50:3), respectively, obtained by UPLC-ESI-TOF-MS.

On the other hand, different assignations were made using UPLC/MS (method 2) for the ESO and AESO molecules. Due to the sample’s complexity, only some TGs were unequivocally identified. The chromatograms corresponding to ETG(50:2) and ETG(50:3) showed that these compounds were not detectable by direct infusion (method 1) and were determined using this methodology instead. Some isomers could also be separated based on their polarity (Figure 9).

**Figure 10:**
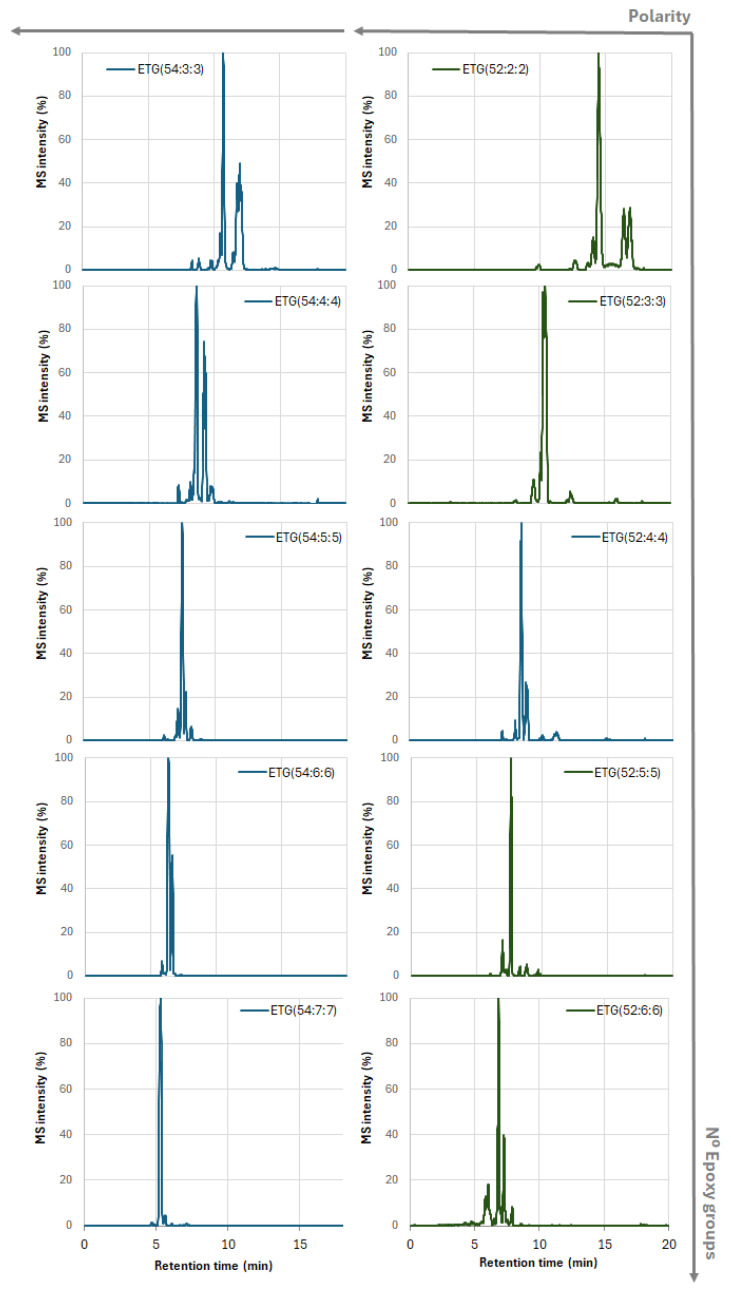
UPLC-ESI-TOF-MS/ESO/Extracted ion-chromatograms at different *m*/*z* corresponding to ETG (54:3);ETG (54:7) and ETG (52:2); ETG (52:6) series.

The series of TGs with 52 and 54 carbons with different degrees of epoxidation were monitored by extracting the ion chromatograms. Thus, various isomers of these ETGs could be separated based on their different polarities (Figure 10). ETG(52:2), ETG(52:3), ETG(52:4), ETG(52:5), and ETG(52:6) were successfully monitored in the following chromatograms (Figure 9). The retention times were according to the higher polarities of more epoxy groups in the TGs.

**Figure 11:**
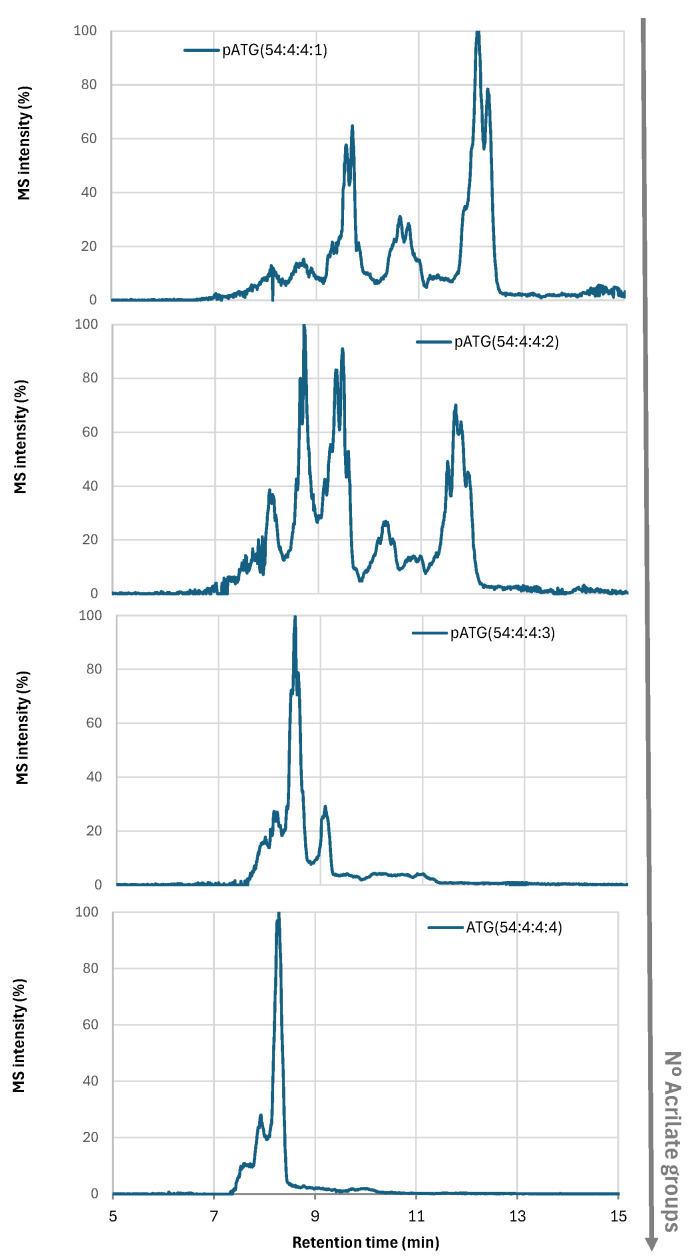
UPLC-ESI-TOF-MS/AESO/Extracted ion-chromatograms at different *m*/*z* corresponding to pATG(54:4:4:1), pATG(54:4:4:2), pATG(54:4:4:3), and ATG(54:4:4:4).

Similar structures could be found in the AESO sample, but in this case, the signals corresponded to acrylated groups instead of epoxide rings (Figure 11). It is worth mentioning that chromatographic separation allowed for the separation and detection of numerous isobaric structures of different acrylated TGs based on their polarity.

## 3. Materials and Methods

### 3.1. Materials

#### 3.1.1. SO Epoxidation

The following reactants were used: SO (cold-pressed, 5 liters, Mystic Moments (Madar Corporation Ltd), Fordingbridge, UK Batch #4446101, www.mysticmomentsuk.com URL accessed on 4 February 2024). Glacial acetic acid (HAc; HPLC-grade, Scharlau, Barcelona, Spain Batch #23893408), hydrogen peroxide (H_2_O_2_; 50% *w*/*w*, Scharlau, Batch #24267501), H_2_SO_4,_ (95–98%, Scharlau, Batch #24332404), Na_2_SO_4_ (extra pure, anhydrous, Thermo Scientific, Madrid, Spain Lot: A0449840), NaCl (99.5%, laboratory reagent-grade, Fisher Chemical, Madrid, Spain, Lot: 2222866), and NaHCO_3_ (99.7%, Scharlau, Batch #22779201).

#### 3.1.2. Partial Acrylation of the Epoxidized Intermediates

Partially epoxidized samples were synthetized using AA (extra pure, stabilized, Thermo Scientific, Lot: A0459748) and hydroquinone (99.5%, Acros Organics, Madrid Spain Lot: A0346098). Solvents and purification reagents were the same as previously described.

### 3.2. Methods

#### 3.2.1. Synthesis of the Epoxidized Intermediates

The epoxidation of SO (129.3 g I_2_/100 g) was previously described elsewhere [12,22]. Reactions were conducted using SO and H_2_SO_4_ as the catalyst. Reactions were started by adding 100 g of SO to an isothermal jacketed reactor, which was mechanically stirred and provided with a reflux condenser. The oil was kept stirring at 400 rpm until it reached the desired reaction temperature (65 °C). Then, 0.3 moles of acetic acid per mol of double bonds of SO were added. A solution of 1.25 mol of H_2_O_2_ per mol of double bonds of the oil and sulfuric acid (2% *w*/*w*) was added drop by drop by means of an HPLC pump with a constant flow of 1 mL/min. The reaction mixture was maintained at constant temperature for 3 h.

To purify the reaction product, it was transferred to 250 mL PE centrifuge tubes and then centrifuged at 3000 rpm for 10 min. The oily phase was recovered and washed 4 times with warm brine solution (60 °C). No organic solvent was used to dissolve the epoxidized oil and favorize the water extraction. This positively resulted in saving costs and reduced residue generation.

When neutral pH of the washing water was achieved, the product was dried and clarified by adding Na_2_SO_4_. Next, it was filtered and further dried in a rotavapor at 45–50 °C.

#### 3.2.2. Production of the Acrylated Epoxidized Monomers

Hydroquinone (70 mg) was dissolved at 80 °C for more than 10 min in a reactor that contained 70 g of the epoxidized oil, then 21.9 mL of AA was added slowly for 1 h. Then, the reaction was heated to 120 °C and kept at this temperature for 3 h and finally cooled down.

The acrylated product was purified with an equal volume of ethyl acetate as the organic phase, which was added to the reactor. Next, it was neutralized with NaHCO_3_ (1% aq.) until the bubbling of CO_2_ stopped. The upper organic phase was separated in 250 mL plastic tubes that were centrifuged at 3000 rpm for 10 min. The white precipitate (presumably sodium polyacrylate) that formed in the interphase was discarded. The washing and centrifugation steps were repeated with NaHCO_3_ (1%) and NaCl (sat.) until neutrality. The organic solution was dried with Na_2_SO_4_ salt, filtered, and the solvent was evaporated on a rotavapor at 45–50 °C.

### 3.3. Characterization

#### 3.3.1. Oxirane Content COOe

It was determined according to the ASTM D1652-04 standard [23]. This process determined the percentage of epoxide groups in an epoxidized intermediate sample and in the final acrylated product through the titration of a standardized 0.1 N perchloric acid solution in glacial acetic acid with a tetraethyl ammonium bromide solution in acetic acid, which includes the sample for analysis.

Likewise, the oxirane yield of the epoxidized intermediate was calculated from the ratio between the experimental oxirane content COO_exp_ and the theoretical oil maximum oxirane content of the oil, according to the following formula [12]:

(7) $$Y_{\mathrm{COO}}\left(\%\right)=\frac{{\mathrm{COO}}_{\exp}}{{\mathrm{COO}}_{\max}}\cdot 100$$

Whereas COO_exp_ was measured empirically, COO_max_ is the theoretical maximum oxirane content of the SO, which was calculated as follows:

(8) COOmax=IV02·Ai100+IV02·Ai·A0·A0·100

A_i_ is the atomic weight of iodine, 126.9; and A_0_ is that of O, 16.0.

#### 3.3.2. Iodine Value (IV)

IV quantifies the unsaturation degree of SO. It was determined using Wij’s method [24]. It is based on the iodometric titration of the remaining iodine content after the reaction with an appropriate reagent.

(9) $$\mathrm{IV}=\frac{12.69\left(V_{b}-V_{s}\right)\cdot N}{W}$$

where (V_b_ − V_s_) represents the difference, in mL, of the sodium thiosulfate solution required for the blank and the sample, respectively. N stands for the normality of the sodium thiosulfate solution in eq/L. The value 12.69 serves as the conversion factor from meq of sodium thiosulfate to grams of iodine (considering that the molecular weight of iodine is 126.9 g/mol). Lastly, W represents the weight of the sample in grams.

#### 3.3.3. FTIR

A Bruker Alpha Platinum ATR spectrometer was used, which collects spectra at a resolution of 4 cm^−1^ in absorbance mode within the range of 400–4000 cm^−1^. For each analysis, a total of 24 scans were carried out, respectively, and they were analyzed using OPUS 6.5 software.

#### 3.3.4. ^1^H NMR

It was used to estimate the acrylation degree of the monomers, which was obtained by the integration of the peak areas referenced to stable protons considered as reference peaks. A Bruker Avance 400 MHz spectrometer equipped with a QNP z gradient probe was used at room temperature. The signals obtained were represented in parts per million (ppm) in relation to the internal standard tetramethylsilane. The spin multiplicity was expressed by s = singlet, d = doublet, t = triplet, q = quartet, and m = multiplet. The analysis was performed after dissolving the acrylated samples in deuterated chloroform (40 mg/mL).

#### 3.3.5. Mass Spectrometry Analysis

This was carried out to determine the position/distribution of the acrylated groups in the TG molecules. Two different approaches were adopted:

Method 1: Direct sample infusion and mass spectrometry detection with an electrospray source and high-resolution time-of-flight analyzer (DI-ESI-TOF-MS).

Mass analyses were carried out on an ESI-QTOF, Synapt XS (Waters, Mildford, MA, USA). All instrument parameters were optimized to obtain the best signal for TGs and their derivatives. Thus, the capillary and cone voltages were set at 700 and 50 V, the nebulizer and cone gas flow rates were 700 and 150 L/h, and the nebulizer and source temperatures were set at 350 and 150 °C, respectively.

The equipment was calibrated externally and prior to analysis using a dilution of sodium iodide at 1 μg/mL in water/isopropanol (1:1). In addition, during the measurements, a dissolution of leucine enkephalin (200 ng/mL in water/isopropanol 1:1) was introduced in parallel in the source (lock mass) in order to adjust the measurements during the analyses and to achieve accuracies below 5 ppm (high resolution—accurate mass).

All samples were diluted to 100 μg/mL in methanol and 1 μL was injected into the equipment using an FNT-type automatic injector (Acquity, Waters, Mildford, MA, USA) and a mobile phase of 10 mM ammonium formiate in water and ACN.

The acquisition time was 30 sec in the mass ranges *m*/*z* 800–1200 and 800–1500 (acrylates). All data obtained were processed using Masslynx v4.2 software (Waters, Mildford, MA, USA) and signal assignment was performed by monitoring mainly the [M + NH4]^+^ adduct.

Method 2: Ultra-high-performance liquid chromatography and mass spectrometry detection with an electrospray source and high-resolution time-of-flight analyzer (UHPLC/ESI-TOF-MS).

Before detection by mass spectrometry, the samples were separated by liquid chromatography using Acquity Premier-type equipment equipped with a BEH C18-type column of dimensions 100 × 2.1 mm and particle size of 1.7 um (Waters, Milford, MA, USA).

A gradient was employed for the chromatographic separation using a mixture of water with 50 mM ammonium formiate (A) and ACN (B) as the mobile phases, as indicated in Table 4:

The column temperature was set at 30 °C, and the injection volume was 1 μL. The total analysis time was 20 min. The detection was carried out with using ESI-QTOF Synapt XS equipment (Waters, Milford, MA, USA) with the use of mass spectrometry. All instrumental parameters were optimized to obtain the best signal for TGs and their derivatives. To achieve this, the capillary voltage and cone voltage were firmly set to 700 and 50 V, respectively, while the nebulization and cone gas flow rates were firmly set at 700 and 150 L/h, respectively, and the nebulization and source temperatures were set at 350 °C. The equipment was externally calibrated using a NaI solution with a concentration of 1 μg/mL in a water–isopropanol solution at a ratio of 1:1. During measurements, a leucine enkephalin solution with a concentration of 200 μg/mL in a water–isopropanol solution in a 1:1 ratio was introduced in parallel into the source (lock mass). The acquisition was performed in positive mode within the precise mass ranges of *m*/*z* 800–1200 and 800–1500 (acrylates). All data obtained were processed using Masslynx v4.2 software (Waters, Milford, MA, USA), and the signal assignment was performed by monitoring the [M + NH4]^+^ adduct.

## 4. Conclusions

A novel methodology based on electrospray ionization techniques (UPLC/ESI-TOF-MS) was developed to accurately analyze the acrylated species formed during the reaction between ESO and AA. These techniques can effectively differentiate between isomers with equivalent mass but different spatial configurations as a function of their polarity and their affinity to the chromatographic column. The characterization and quantification of these structures is a complex task that requires advanced MS/MS characterization to ensure that each isomer is unequivocally assigned. These methods are very useful for controlling the functionalities (epoxide groups and acrylic bonds) of modified triglycerides, which can affect the properties of acrylate polymers, such as the crosslinking degree, Tg, or durability.

## Figures and Tables

**Table 1:** FTIR characteristic peaks of SO and ESO.

Wavenumber (cm^−1^)	Functional Group
3000	=C–H stretching
2925	CH_2_ stretching
2860	CH_3_ stretching
1740	C=O asymmetric stretching
1650	C=C stretching
1460	CH_2_ scissors
1230	C=C–C–O stretching
1160	C–O asymmetric axial stretching
823	C–O–C asymmetric epoxide stretching
723	CH_2_ Rocking

**Table 2:** Summary of principal new peaks that appeared in the AESO molecules’ spectra.

Wavenumber (cm^−1^)	Functional Group
3400–3500	–OH stretching
1721	C=O acrylate stretching
1635	CH_2_=CH– acrylate stretching
1619	CH_2_=CH–COO– tension
1405	CH_2_=CH- acrylate flexion
1050	=C–H bending (twisting)
1000	–C–O-C stretching
810	–HC=CH– out of plane bending vibration

**Table 3:** Assignment of MS signals and their absolute and relative intensities. Series (54:4).

Lipid	Mass Intensity (a.u.)	Relative Mass Intensity (%)	Acrylic Groups
ETG(54:4:4:0)	469,000	2.8	0
pATG(54:4:4:1)	2,740,000	16.4	1
pATG(54:4:4:2)	5,520,000	33.1	2
pATG(54:4:4:3)	4,800,000	28.8	3
ATG(54:4:4:4)	3,140,000	18.8	4

**Table 4:** Mixtures of A and B used for chromatographic separation.

Time (min)	Flow (mL/min)	% A	% B
0	0.3	30	70
1	0.3	30	70
7	0.3	1	99
17	0.3	1	99
17.5	0.3	30	70
20	0.3	30	70

## Data Availability

The data presented in this study are available upon request from the corresponding authors.
